# Supplementary material for: Development of a High-Quality ELISA Method for Dinotefuran Based on a Novel and Newly-Designed Antigen
Source: Molecules. 2019 Jul 2;24(13):2426. doi: 10.3390/molecules24132426 (PMC6651010; doi:10.3390/molecules24132426)
Supplement: Supplementary file 1 [file molecules-24-02426-s001.zip › NMR/NMR.docx]

^1^H NMR (400 MHz, CD_3_OD) δ 1.62-1.72 (3H, m, H-3, 4), 2.02-2.15 (4H, m, H-14, 15), 2.58(3H, s, H-10), 2.93 (1H, d, 10.52 Hz, H-6a), 3.19 (1H, d, 7.48Hz, H-6b), 3.53-3.58 (2H, m, H-2), 3.75-3.91(2H, m, H-5). ^13^C NMR (100 MHz, CDCl_3_) δ 18.83 (C-10), 27.11 (C-15), 28.43 (C-14), 29.21 (C-4), 37.02 (C-6), 43.71 (C-3), 67.20 (C-5), 70.54 (C-2), 174.75 (C-13), 177.76 (C-17).
