# Supplementary figures and images for: Development of a High-Quality ELISA Method for Dinotefuran Based on a Novel and Newly-Designed Antigen
Source: Molecules. 2019 Jul 2;24(13):2426. doi: 10.3390/molecules24132426 (PMC6651010; doi:10.3390/molecules24132426)

CYJ-2  
CYJ-2

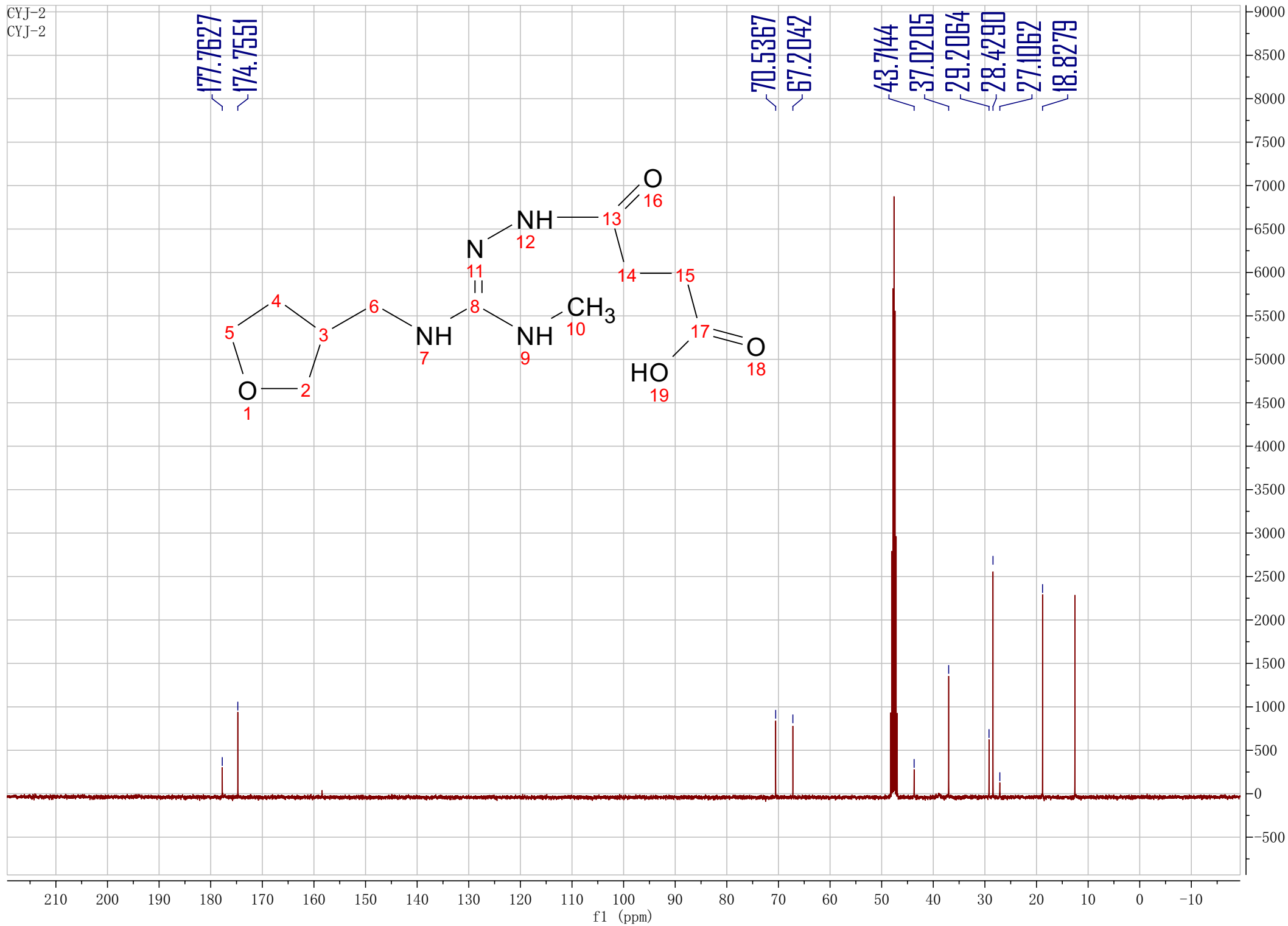

Supplement: Supplementary file 1 [file molecules-24-02426-s001.zip › NMR/NMR-13C.pdf]

201805081  
201805081

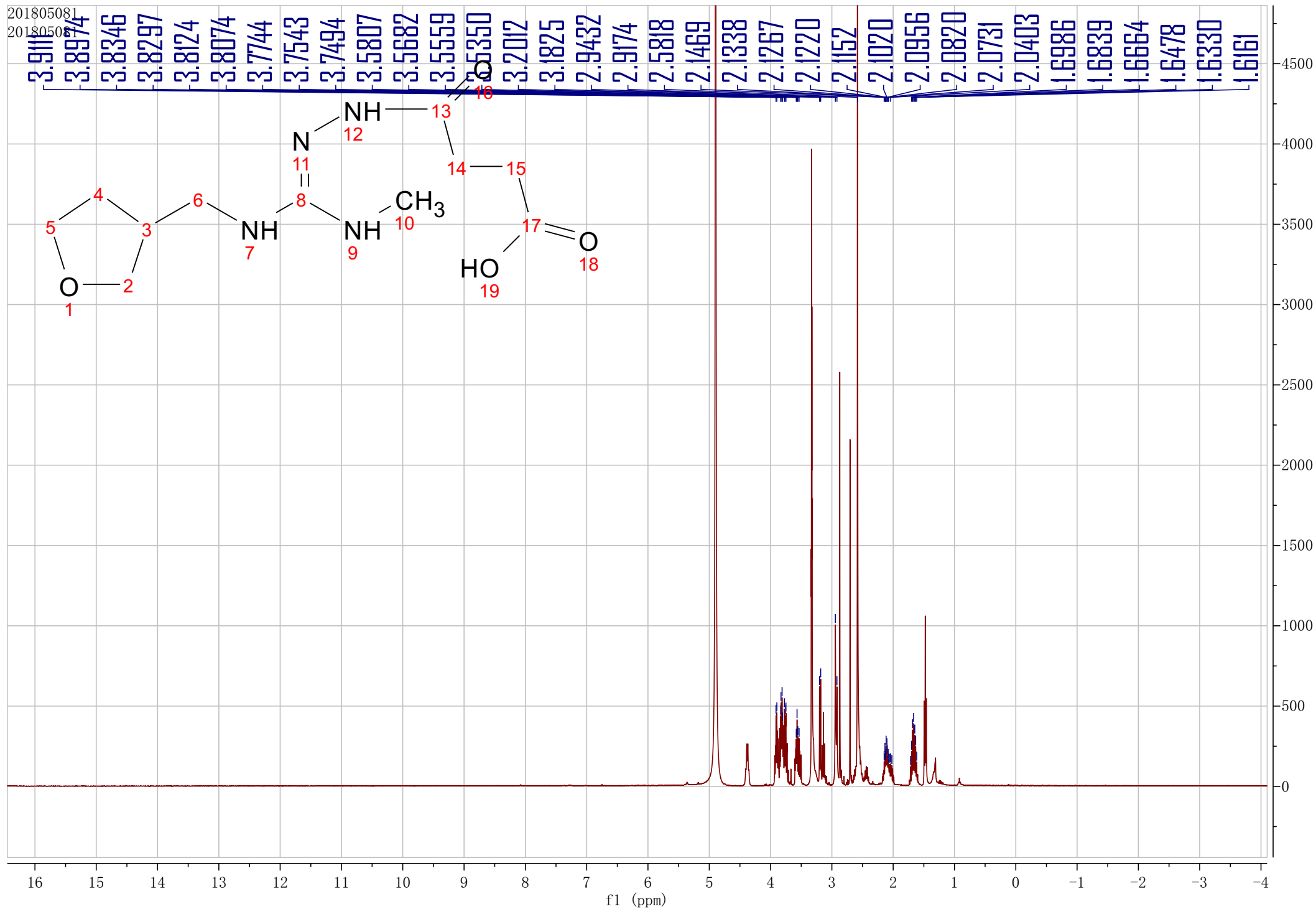

Supplement: Supplementary file 1 [file molecules-24-02426-s001.zip › NMR/NMR-1H.pdf]
